# Styrylpyrazoles: Properties, Synthesis and Transformations

**DOI:** 10.3390/molecules25245886

**Published:** 2020-12-12

**Authors:** Pedro M. O. Gomes, Pedro M. S. Ouro, Artur M. S. Silva, Vera L. M. Silva

**Affiliations:** LAQV-REQUIMTE, Department of Chemistry, University of Aveiro, 3810-193 Aveiro, Portugal; pm.gomes@ua.pt (P.M.O.G.); pedroouro@ua.pt (P.M.S.O.)

**Keywords:** pyrazoles, styrylpyrazoles, biological activity, organic synthesis, reactivity

## Abstract

The pyrazole nucleus and its reduced forms, pyrazolines and pyrazolidine, are privileged scaffolds in medicinal chemistry due to their remarkable biological activities. A huge number of pyrazole derivatives have been studied and reported over time. This review article gives an overview of pyrazole derivatives that contain a styryl (2-arylvinyl) group linked in different positions of the pyrazole backbone. Although there are studies on the synthesis of styrylpyrazoles dating back to the 1970s and even earlier, this type of compound has rarely been studied. This timely review intends to summarize the properties, biological activity, methods of synthesis and transformation of styrylpyrazoles; thus, highlighting the interest and huge potential for application of this kind of compound.

## 1. Introduction

**Figure 1:**
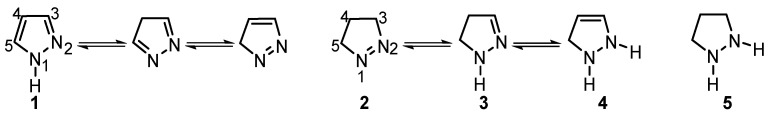
Chemical structures and numbering of pyrazole **1**, dihydropyrazole (pyrazoline) tautomers **2**–**4** and pyrazolidine **5**.

The pyrazole (1*H*-pyrazole, **1**) (Figure 1) is an aromatic five-membered heterocyclic ring constituted by three carbons and two adjacent nitrogen atoms, located at 1- and 2-positions [1]. *N*-unsubstituted pyrazoles may present three identical and non-separable tautomers, due to rapid interconversion in solution, and it is usually impossible to unequivocally assign the resonances of its proton-nuclear magnetic resonance (^1^H-NMR) spectrum. Three partially reduced forms may also exist: 1-pyrazolines **2**, 2-pyrazolines **3** and 3-pyrazolines **4**, and a fully reduced form known as pyrazolidine **5** (Figure 1) [1,2].

Pyrazole and its derivatives occupy a prime place in medicinal chemistry because they are present in several drugs with real medicinal applications, such as celecoxib (Celebrex^®^), sildenafil (Viagra^®^), rimonabant, lonazolac, fomepizole, penthiopyrad, doramapimod, sulfaphenazole and in several remarkable compounds with a wide range of pharmacological activities, namely anticancer [3], analgesic, anti-inflammatory and antioxidant [4,5,6,7], antibacterial and antifungal [8,9] antipyretic, antidepressant and anticonvulsant [10,11], antidiabetic [12] and cannabinoid activities [13,14], among others [15,16,17,18,19,20,21]. Along with the medicinal applications, pyrazoles are also useful as agrochemicals, such as herbicides, fungicides, and insecticides [22] and possess other applications, such as dyestuffs, sunscreen materials [23,24], analytical reagents and pluripotent ligands in coordination chemistry [25,26]. For instance, polyaromatic pyrazoles possess important biological, photophysical, optical and electronic properties [10,27,28,29].

This review is focused on a particular type of pyrazoles, the styrylpyrazoles (or styryl-1*H*-pyrazoles), which present a styryl (2-arylvinyl) group at one or more positions of the pyrazole nucleus. It is intended to give an overview of the styrylpyrazoles chemistry reported in the literature, including their properties and biological activity, since the 1970s to the present date. Despite the biological activities of styrylpyrazoles [12,14], their interesting physicochemical properties, such as tautomerism, isomerism [30,31] and conjugation extension, due to the presence of the 2-arylvinyl group, as well as the rich chemistry related to their synthesis and transformation, this type of pyrazole has rarely been studied, as can be seen from the low number of publications in a very large time span covered by this review. In our group, we have been working with styrylpyrazoles for a long time. Throughout this review, we intend to describe the properties and methods of synthesis of these compounds, and to show their great potential for application in different fields, from medicine to materials chemistry, including their use as key templates for transformation into other valuable compounds. The articles will be presented based on the molecules’ structure, in particular, the position of the styryl group in the pyrazole scaffold.

## 2. Properties of Styrylpyrazoles

**Figure 2:**
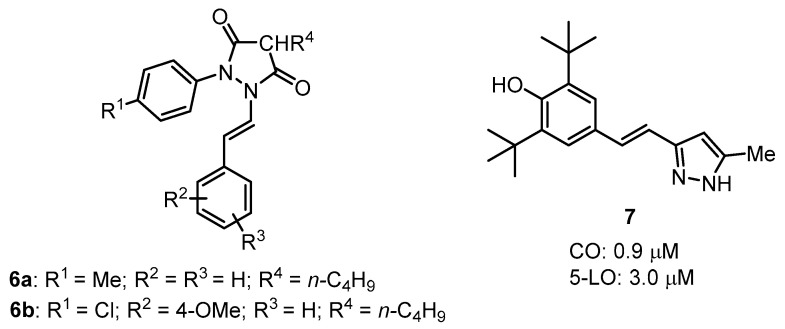
Styrylpyrazole derivatives **6** and **7** presenting anti-inflammatory activity [32,33].

The pharmacological activity of styrylpyrazoles and related compounds has been known for a long time. A study published in 1970 described the anti-inflammatory activity of 1-phenyl-2-styryl-3,5-dioxopirazolidines (**6**). Among these compounds, derivatives **6a** (R^1^ = Me, R^2^ = R^3^ = H, R^4^ = *n*-C_4_H_9_) and **6b** (R^1^ = Cl, R^2^ = 4-OMe, R^3^ = H, R^4^ = *n*-C_4_H_9_) (Figure 2) were more active than phenylbutazone or oxyphenbutazone in the carrageenin-induced foot edema test in rats [32]. It was found that a *p*-methylphenyl ring increased the intrinsic activity in general, while a *p*-chlorophenyl substituent decreases the activity, except for compound **6b**. The *p*-methoxy or 3,4-methylenedioxy substituents of the styryl group tend to increase both toxicity and inhibitory activity, while *p*-alkyl substituents (Me or *i*-Pr) have the opposite effect. 

Styrylpyrazoles were also found to act as dual inhibitors of 5-lipoxygenase (5-LO) and cyclooxygenase (CO) in rat basophilic leukemia cells. In particular, compound **7** (Figure 2) exhibited oral activity in various models of inflammation and, most importantly, is devoid of ulcerogenic potential [33].

**Figure 3:**
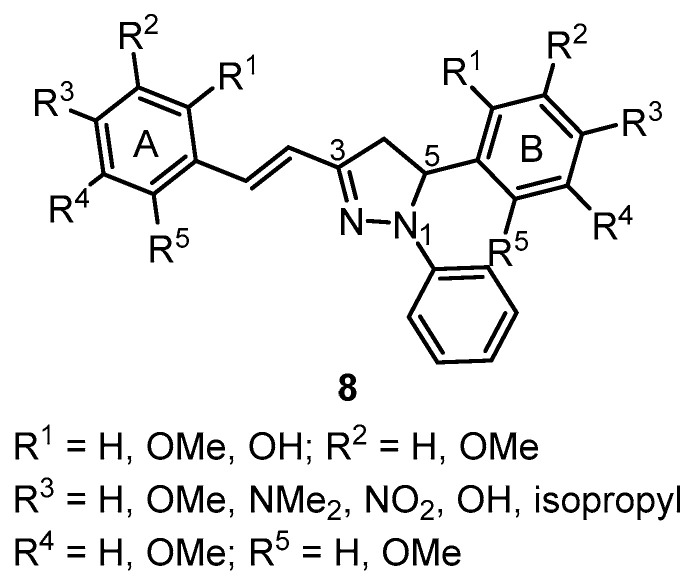
3-Styryl-2-pyrazolines **8** presenting antibacterial and antifungal activity [34].

Nauduri et al. studied the antibacterial activity of 1,5-diphenyl-3-styryl-2-pyrazolines **8**, with different substituents (OMe, NMe_2_, NO_2_, OH and isopropyl) (Figure 3), towards Gram-positive and Gram-negative bacteria and found little variation in the geometric means of minimum inhibitory concentrations (MIC) [34]. The derivatives containing NMe_2_, NO_2_ and OMe in the *p*-position of A and B aromatic rings showed the highest activity (MIC = 3.55–6.56 μg/mL and 3.30–6.27 μg/mL against Gram-positive and Gram-negative bacteria, respectively). This fact was attributed to the strong electronic-driving nature of the substituents (either as electron-donating or electron-withdrawing substituents) and due to a more lipophilic nature (for the NMe_2_). Low substitution in the aromatic ring also favored the entry of the compound in the bacteria through passive diffusion. They have compared the activity of these compounds with that of 1,3-diphenyl-5-styryl-2-pyrazolines, which showed lower activities than the compounds of the 3-styryl series. Moreover, the 1,5-diphenyl-3-styryl-2-pyrazolines **8** bearing *o*- or *p*-hydroxyphenyl rings showed superior antimycotic (antifungal) activity relative to the other derivatives (MIC = 11.3–24.8 μg/mL and 12.16–13.6 μg/mL against fungi and yeast, respectively). The extended electronic structure of the 3-styryl compared with the 5-styryl derivatives may explain the lower MIC values for the compounds of the 3-styryl series [34].

**Figure 4:**
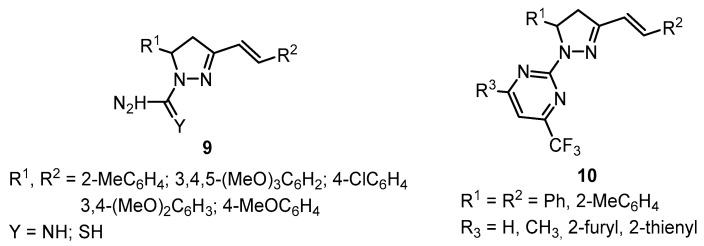
5-Aryl-3-styryl-1-carboxamidino-1*H*-pyrazole derivatives presenting antioxidant and antimicrobial activity **9** and **10** [35].

In the 2,2-diphenyl-1-picrylhydrazyl (DPPH) and horseradish peroxidase (HRP)/luminol/H_2_O_2_ chemiluminescence assays, 5-aryl-3-styryl-1-carboxamidino-1*H*-pyrazole derivative **9** showed higher antioxidant activity (half maximal inhibitory concentration (IC_50_) 45.17–217.22 μg/mL, in DPPH assay) than 2-(5-aryl-3-styryl-1*H*-pyrazol-1-yl)-4-trifluoromethylpyrimidines **10** (Figure 4). The antioxidant activity is dependent on the concentration and type of substituents in each compound, and resonance stabilization. For instance, the presence of SH at Y position strongly enhances antioxidant activity. Some derivatives of compound **9** showed antimicrobial activity against bacteria *Salmonella typhi*, *Staphylococcus aureus* and *Streptococcus pneumoniae*, with inhibition between 1.95 to 15.625 mg/mL. The antimicrobial activity is also affected by the substituent [35].

**Figure 5:**
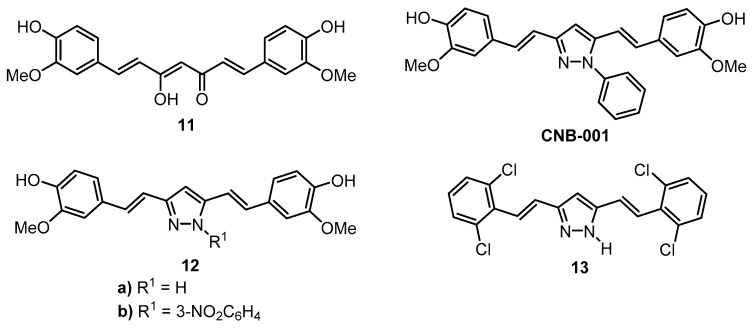
Structures of curcumin (**11**) and 3,5-bis(styryl)pyrazoles **CNB**-**001**, **12** and **13**, presenting antioxidant, antimalarial, cytotoxic and antiproliferative activities [36,37,38,39,40,41,42,43,44].

Furthermore, 3,5-bis(styryl)pyrazoles **12**, curcumin (**11**) analogues (Figure 5), are well-known for their antioxidant activity. Some derivatives showed superior activity to that of curcumin in DPPH, ferric reducing antioxidant power (FRAP), and β-carotene bleaching assays [36]. For instance, curcumin (**11**) showed lower inhibition of DPPH^•^ (102 mmol), (half maximal effective concentration (EC_50_) = 40 ± 0.06 μmol)) compared with 3,5-bis(4-hydroxy-3-methoxystyryl)pyrazole (**12a**) (EC_50_ = 14 ± 0.18 μmol). The presence of hydroxy and methoxy groups on the terminal phenyl rings is considered benefic for the antioxidant capacity. Moreover, the 3,5-bis(styryl)pyrazole **CNB-001** has shown interesting results in studies related to neuroprotection and Alzheimer’s disease, and was able to restore membrane homeostasis disrupted after brain trauma, being a promising compound for therapeutic use in the treatment of Parkinson’s disease [37]. In combination with tissue plasminogen activator, **CNB**-**001** is efficient in the treatment of stroke [38]. Compound **12a** also acted as inhibitor of β-amyloid secretion and of protein kinases involved in neuronal excitotoxicity [39]. Some pyrazoles that are analogues of **12a** displayed good inhibition of γ-secretase activity, tau aggregation, and/or affinity to fibrillar Aβ42 aggregates [40].

Other properties have been attributed to 3,5-bis(styryl)pyrazoles such as antimalarial, antimycobacterial, antiangiogenic, cytotoxic and antiproliferative activities [39,41,42]. As an example, 3,5-bis(styryl)pyrazoles **12a**,**b** showed more effective inhibition of chloroquine-sensitive *Plasmodium falciparum* (IC_50_ = 0.48 ± 0.04 μM and 0.87 ± 0.07 μM) and chloroquine-resistant *Plasmodium falciparum* (IC_50_ = 0.45 ± 0.07 μM and 0.89 ± 0.10 μM), respectively, than curcumin (IC_50_ = 3.25 ± 0.6 μM and 4.21 ± 0.8 μM) [41]. 

Several studies have highlighted the antiproliferative activity of **12a** in PC3 cells, with growth inhibitory concentration (GI_50_) values of 5.6 μM [43] and in breast cancer cell lines, MCF-7 and SKBR3, with GI_50_ values of 4.19 μM and 0.25 μM, respectively [42]. Liao and coworkers have also shown the antiproliferative activity of 3,5-bis(styryl)pyrazoles **12a** and **13** in the androgen-independent PC3 prostate cancer cell line with GI_50_ values in the low micromolar range. In particular, compound **13** caused significant effects on the PC3 cell cycle, inducing cell death and binding to tubulin (Kd 0.4 ± 0.1 μM), inhibiting tubulin polymerization in vitro, being competitive with paclitaxel for binding to tubulin, and leading to microtubule depolymerization in PC3 cells. Therefore, **13** was considered as a lead compound for the treatment of castration-resistant prostate cancer (CRPC) [44].

**Figure 6:**
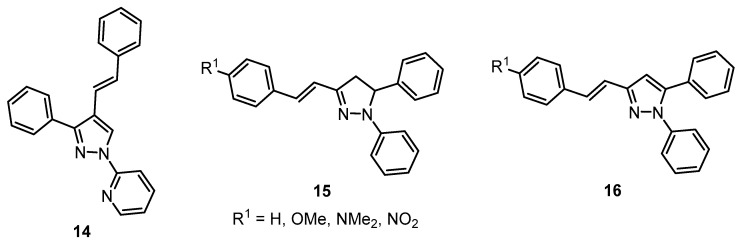
Styrylpyrazoles **14**–**16** presenting relevant photophysical properties [45,48,49,50,51,52].

In addition to pharmacological activities, 4-styrylpyrazoles have shown interesting photophysical properties. In particular, 1-(2-pyridyl)-4-styrylpyrazoles (PSPs) substituted at position 3 with donor or acceptor aryl groups have shown strong blue-light emissions with high quantum yields (up to 66%), due to intramolecular charge transfer (ICT) phenomena [45]. The 3-phenyl-1-(2-pyridyl)-4-styrylpyrazole **14** (Figure 6) was studied as a turn-off fluorescent probe in metal ion sensing, showing a high selectivity to Hg^2+^ (Limit of detection (LOD) = 3.1 × 10^−7^ M) in a process that could be reversed with ethylenediamine.

Although pyrazoles are rare in nature, due to the difficulty of living organisms to construct the N–N bond [46,47], a styrylpyrazole natural derivative, (*E*)-1,5-diphenyl-3-styryl-2-pyrazoline (DSDP) **15** (also known as (*E*)-1,5-diphenyl-3-styryl-4,5-dihydro-1*H*-pyrazole) (Figure 6), was isolated from the aerial parts of *Euphorbia guyoniana* [48]. In 2017, Kundu et al. unveiled the binding interaction of DSDP **15** with the calf thymus DNA (ctDNA) [49]. By performing steady state and time resolved spectroscopic measurements, competitive binding studies, circular dichroism and a DNA helix melting study they unequivocally demonstrated that DSDP binds with the ctDNA through groove binding mode with no conformational change. These findings were also supported by molecular docking simulation.

Later, the same authors reported the strong binding interactions of DSDP with two serum transport proteins, human serum albumin (HSA) and bovine serum albumin (BSA) [50]. Exploiting multi-spectroscopic techniques together with in silico molecular docking simulation, they have demonstrated that DSDP is a potent fluorophore. It binds with both proteins with the formation of a 1:2 protein–probe complex at a lower protein concentration range and a 1:1 complex at a higher protein concentration range. The calculated binding constants for the 2:1 DSDP-protein complexes were found to be 1.37 × 10^10^ M^−2^ and 1.47 × 10^10^ M^−2^ for HSA and BSA, respectively, while for the 1:1 complexation process, the constants were 1.85 × 10^5^ M^−1^ and 1.73 × 10^5^ M^−1^ for DSDP-HSA and DSDP-BSA systems, respectively. Moreover, they have demonstrated that hydrogen bonding and hydrophobic interactions are the forces primarily responsible for both types of binding. The information gathered from these studies may be useful for the rational design of drugs looking at a greater clinical efficacy.

1,3,5-Trisubstituted pyrazolines, analogues of DSDP, exhibited large fluorescence quantum yields (Φ_f_ = 0.6–0.8), suited for the design of energy-transfer-based fluorescent probes [51]. In DSDP’s structure, two of the aryl rings (phenyl and styryl) are interconnected electronically through the pyrazoline π-system while the 5-phenyl ring is electronically decoupled and can behave as an electron donating receptor for cations or electron deficient centers. The lone pair of electrons on the N1 atom of the pyrazoline ring also takes part in the conjugation, facilitating the ICT process. Thus, DSDP acts as a D-π-A system and the introduction of an electron withdrawing group (such as -CN, -NO_2_, -COOEt) on the styryl moiety can enhance the push–pull D-π-A capacity of DSDP-like molecular systems [52]. However, a drastic modification of the photophysical properties of DSDP is observed upon dehydrogenation of the pyrazoline ring with subsequent formation of the corresponding pyrazole (DSP) **16** (Figure 6). While DSDP gives dual absorption and dual emission bands corresponding to the locally excited (LE) and ICT species, DSP yields single absorption and emission bands for the LE species only. Comparative steady state and time resolved fluorometric studies have revealed that aromatization of the pyrazoline ring completely inhibits the ICT process. These results were also corroborated by quantum chemical calculations [52].

## 3. Synthesis of Styrylpyrazoles

In this section, the methods for the synthesis of styrylpyrazoles are described in a systematic way, based on the compounds’ structure, considering the position of the styryl group in the pyrazole scaffold, starting from C-1 to C-5. Bis(styryl)pyrazoles, substituted at C-3 and C-5, are also considered. Moreover, in each subheading, the methods will be presented by alphabetic order. Single examples of a certain reaction will be considered at the end of each section in the miscellaneous subheading.

### 3.1. Synthesis of 1-Styrylpyrazoles

#### 3.1.1. *N*-Cross-Coupling Reaction of Pyrazoles with Styrylboronic Acid

**Scheme 1:**
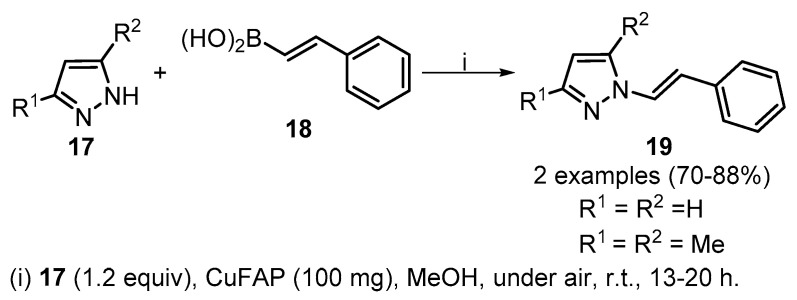
Cross-coupling reactions of pyrazoles **17** with styrylboronic acid **18** to produce (*E*)-1-styrylpyrazoles **19** [53].

In 2010, Kantam et al. synthesized (*E*)-1-styrylpyrazoles in a simple and efficient way, by the cross-coupling reaction of pyrazoles **17** with styrylboronic acid **18** using a recyclable heterogeneous Cu-exchanged fluorapatite (CuFAP) catalyst, under base-free conditions (Scheme 1) [53]. The lower yield (70%) for the coupling with 3,5-dimethyl-1*H*-pyrazole **17** (R^1^ = R^2^ = Me) was attributed to the steric hindrance caused by the methyl groups. When the reaction was performed in the absence of air, no coupled product was obtained, since O_2_ is involved in the oxidation of Cu(I) to Cu(II), which is the active species in the reaction mechanism [53].

#### 3.1.2. *N*-Styrylation of Pyrazoles with Activated and Non-Activated Alkynes

**Scheme 2:**
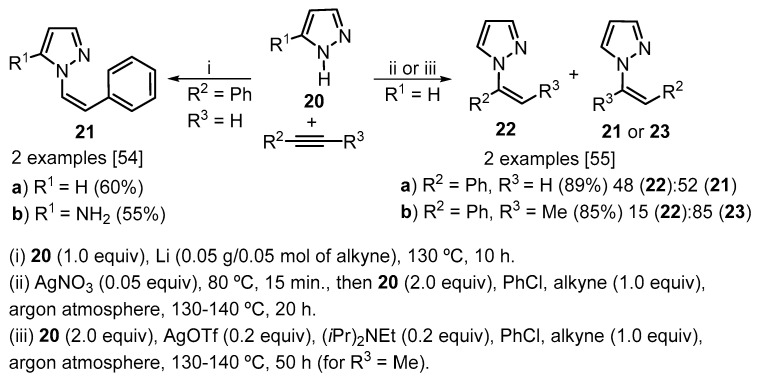
Addition of pyrazole **20** to alkynes to produce (*Z*)-1-styrylpyrazoles **21**–**23** [54,55].

In 1978, Burgeois et al. described a lithium-catalyzed stereospecific and stereoselective addition of the N–H bond of pyrazole **20** to phenylacetylene, as a method to prepare 1-styrylpyrazoles **21** (Scheme 2) [54]. Under such conditions, (*Z*)-1-styryl-1*H*-pyrazole (**21a**) and (*Z*)-5-amino-1-styryl-1*H*-pyrazole (**21b**) were obtained in 60% and 55% yield, respectively.

Tsuchimoto et al. reported a similar single addition of the N–H bond of pyrazoles to phenylacetylene and prop-1-yn-1-ylbenzene in the presence of a silver catalyst (AgNO_3_ or AgOTf) to produce a regioisomeric mixture of 1-substituted pyrazoles **22** and **23** (Scheme 2) [55]. Complete stereoselectivity was achieved, since only the (*Z*)-isomer of 1-styrylpyrazole was formed, indicating that *anti*-addition, via a concerted mechanism, in which pyrazole nitrogen attacks the alkyne from the side opposite to a coordinated Lewis acid (LA), is involved as a key step in the reaction process [55].

**Scheme 3:**
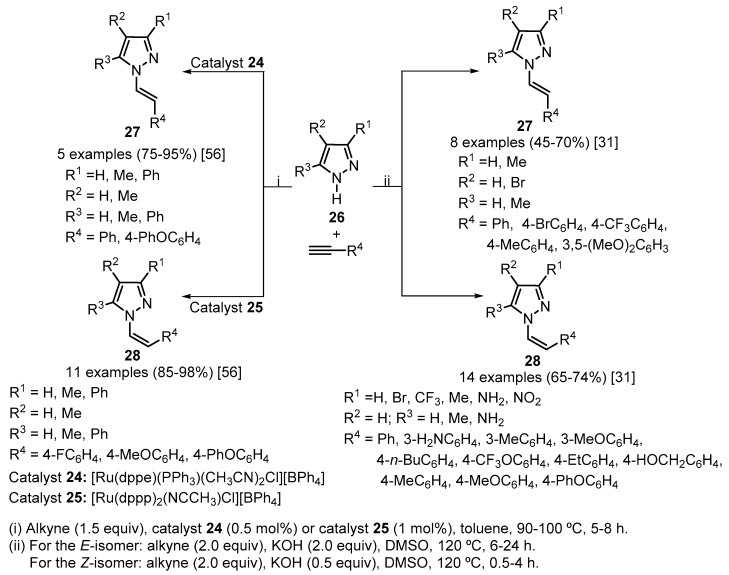
Stereoselective additions of pyrazoles **26** to alkynes to produce (*E*)- and (*Z*)-1-styrylpyrazoles **27** and **28** [31,56].

The role of two ruthenium complexes [Ru(dppe)(PPh_3_)(CH_3_CN)_2_Cl][BPh_4_] (**24**) (dppe = diphenylphosphinoethane) and [Ru(dppp)_2_(NCCH_3_)Cl][BPh_4_] (**25**) (dppp = diphenylphosphinopropane) as efficient catalysts in the regio- and stereoselective addition of pyrazoles **26** to alkynes was described by Das Kumar et al. (Scheme 3) [56]. In general, the addition catalyzed by complex **24** afforded (*E*)-1-styrylpyrazoles **27** while complex **25** produces (*Z*)-1-styrylpyrazoles **28**. Based on density functional theory calculations, the authors have shown that stereoselectivity is dependent upon the ligand environment around the ruthenium center.

Recently, Garg et al. reported a transition-metal-free chemo-, regio-, and stereoselective synthesis of (*Z*)- and (*E*)-1-styrylpyrazoles by the addition of pyrazoles **26** onto functionalized terminal alkynes using a super basic solution of KOH/dimethyl sulfoxide (DMSO) [31]. The nature of the base seems to be crucial for the reaction, which does not occur in the presence of an organic base, such as triethylamine (Et_3_N). The reaction stereoselectivity is governed by time and amount of the base (Scheme 3). Deuterium labeling and control experiments highlighted the role of the KOH/DMSO catalytic system in the *cis*→*trans* isomerization, which was further supported by comparative ^1^H-NMR spectrum studies in DMSO/DMSO-d_6_. This method is of wide scope and several functionalities, such as OH or Me, NH_2,_ present both in the alkyne and pyrazole are well-tolerated [31]. 

### 3.2. Synthesis of 3(5)-Styrylpyrazoles

#### 3.2.1. Cyclocondensation Reactions

**Scheme 4:**
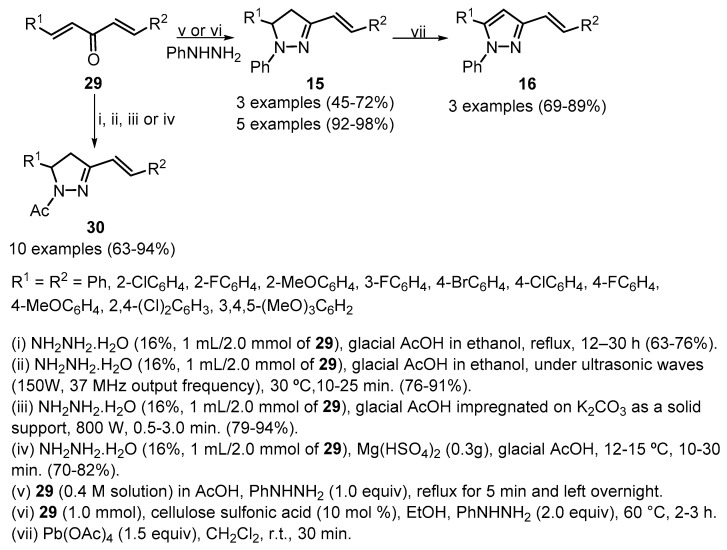
Synthesis of 3-styrylpyrazolines **15** and **30** and 3-styrylpyrazoles **16** [58].

The cyclocondensation of (1*E*,4*E*)-1,5-diarylpenta-1,4-dien-3-one **29** with hydrazine or phenyl hydrazine in glacial acetic acid at reflux [57,58], in the presence of sulfuric acid [59], or cellulose sulfonic acid [60], afforded 5-aryl-3-styryl-4,5-dihydro-1*H*-pyrazoles (pyrazolines) **15** or **30** (Scheme 4). Following a similar approach, Nauduri et al. synthesized a series of 5-aryl-1-phenyl-3-styryl-4,5-dihydro-1*H*-pyrazoles by condensation of **29** analogues with phenyl hydrazine hydrochloride in a mixture of ethanol and chloroform, in the presence of a catalytic amount of concentrated HCl (70–77%) [34]. Pathak et al. studied the reaction of **29** with hydrazine hydrate in glacial acetic acid under conventional reflux, ultrasound, microwave irradiation conditions and using mechanochemical mixing, and isolated 1-acetyl-5-aryl-3-(substituted-styryl)pyrazolines **30**. In turn, pyrazolines **15** were converted into the corresponding pyrazoles by oxidation (dehydrogenation) (Scheme 4). Several oxidant agents can be employed for this transformation; some common examples include lead tetraacetate [58], DMSO in open air [61], MnO_2_ [62], *p*-chloranil [63], 2,3-dichloro-5,6-dicyano-1,4-benzoquinone (DDQ) [64], Pd/C [65]. In 2014, Ananthnag et al. developed a simple and high yielding method for the conversion of (*E*)-3- and 5-styrylpyrazolines into the corresponding pyrazoles via iron(III) catalyzed aerobic oxidative aromatization [65]. The use of FeCl_3_ as catalyst makes the reaction greener and more economical. 

**Scheme 5:**
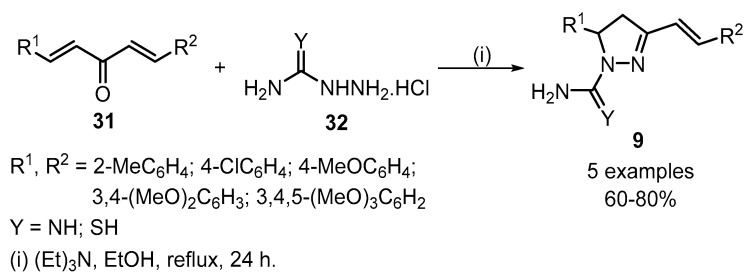
Synthesis of 5-aryl-1-carboxamidino-3-styryl-4,5-dihydro-1*H*-pyrazoles **9** [35,66,67].

Gressler et al. described the [3 + 2] cyclocondensation of 1,5-diarylpenta-1,4-dien-3-ones **31** with aminoguanidine hydrochloride **32** in ethanol, in the presence of triethylamine, as a method to prepare 5-aryl-1-carboxamidino-3-styryl-4,5-dihydro-1*H*-pyrazoles **9** (Scheme 5) [35,66,67].

**Scheme 6:**
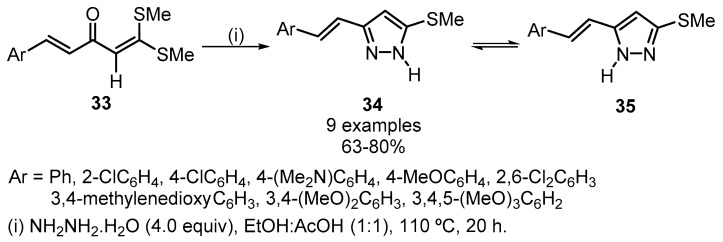
Reaction of oxoketene dithioacetals **33** with hydrazine hydrate to synthesize (*E*)-3(5)-styrylpyrazoles **34** [68].

In 1991, Purkayastha et al. reported the reaction of α-oxoketene dithioacetal **33** with hydrazine hydrate in ethanol, in the presence of acetic acid, as a method to exclusively obtain (*E*)-3(5)-styrylpyrazoles (**34⇌35**) (Scheme 6). They have shown that acidic medium is required for the regioselective formation of **34**, otherwise, in neutral conditions, other products are formed [68]. The formation of styrylpyrazoles **34**, in acidic conditions, was explained through the attack of hydrazine on protonated **33** at the β-carbon with a positive charge, which is stabilized by the two methylthio groups. In fact, the (*E*)-3(5)-styrylpyrazole **34a** (Ar = Ph) was isolated as the sole product (79%) when **33a** (Ar = Ph) reacted with hydrazine hydrate in ethanol:acetic acid (1:1) mixture. Other cinnamoyl oxoketene dithioacetals similarly reacted in these conditions affording a series of (*E*)-3(5)-styrylpyrazoles. This method is also suitable for the synthesis of 3-(4-arylbuta-1,3-dienyl)pyrazoles and 3-(6-arylhexa-1,3,5-trienyl)pyrazoles starting from the appropriate dienoyl- and trienoyl oxoketene dithioacetals, and has high practical utility, since the introduction of such enyl side-chains in the preformed pyrazole is not possible [68].

**Scheme 7:**
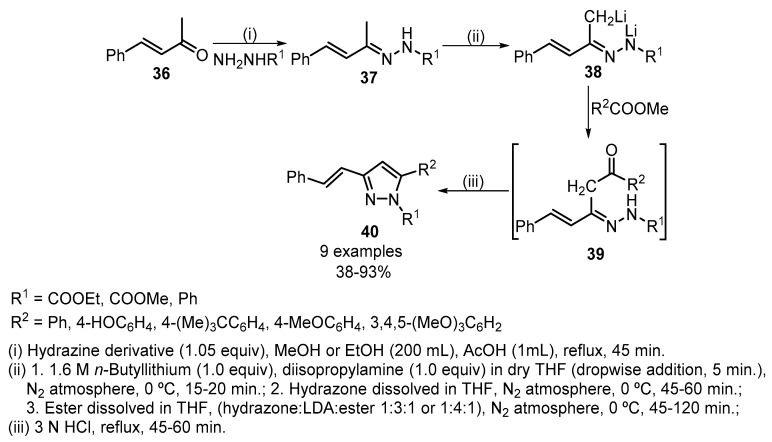
Reaction of benzalacetone **36** with hydrazines to produce hydrazones **37** and their transformation into (*E*)-3-styrylpyrazoles **40** [69].

In 2001, Pastine et al. reported the synthesis of (*E*)-3-styrylpyrazoles starting from benzalacetone **36**, which was converted into the corresponding hydrazones **37** by reaction with hydrazine derivatives. Then, compounds **37** were deprotonated with an excess of lithium diisopropylamide (LDA), and the resulting dilithiated intermediates **38** were condensed at the carbanion center with a variety of substituted benzoate esters, such as methyl benzoate, methyl 4-*t*-butylbenzoate, (lithiated) methyl 4-hydroxybenzoate, or methyl 3,4,5-trimethoxybenzoate affording **39**. After acid cyclization of **39** with 3 N hydrochloric acid, the 3-styrylpyrazoles **40** were isolated (Scheme 7) [69].

**Scheme 8:**
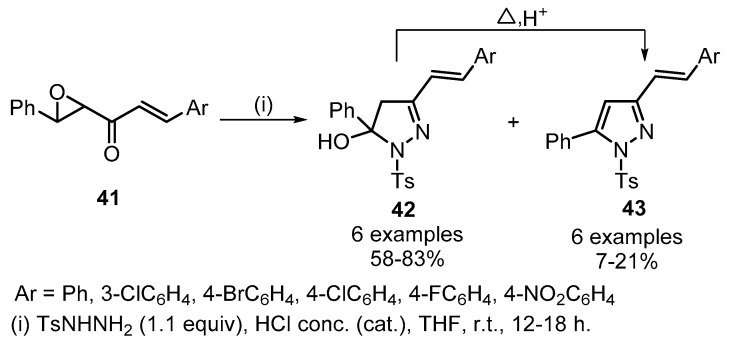
Synthesis of (*E*)-5-hydroxy-5-phenyl-3-styryl-1-tosyl-2-pyrazolines (**42**) and (*E*)-5-phenyl-3-styryl-1-tosyl-1*H*-pyrazoles (**43**) [70].

The reaction of (*E*)-3-aryl-1-(3-phenyloxiran-2-yl)-prop-2-en-1-ones (**41**) with tosyl hydrazine under acid catalysis afforded (*E*)-5-hydroxy-5-phenyl-3-styryl-1-tosyl-2-pyrazolines (**42**) (Scheme 8) [70]. Then, (*E*)-5-phenyl-3-styryl-1-tosyl-1*H*-pyrazoles (**43**), which are formed due to dehydration of 5-hydroxypyrazolines **42**, were also isolated, as by-products, by chromatography, after crystallization of the major products. 

**Scheme 9:**
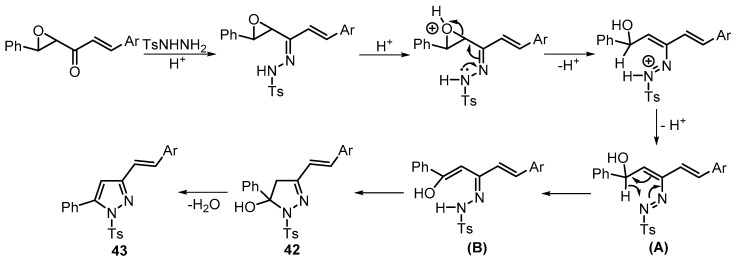
Mechanism of formation of (*E*)-3-styrylpyrazolines **42** and (*E*)-3-styrylpyrazoles **43** [70].

The formation of pyrazolines **42** occurred through oxirane ring-opening at the α-carbon, followed by the rearrangement of the azadiene intermediate (**A**) through a hydride [1,5] sigmatropic shift to 1,3-diketone monohydrazone (**B**, Scheme 9). Finally, the intramolecular cyclization led to 5-hydroxypyrazolines **42**. These compounds are only stable if they have an electron-withdrawing group, such as 1-acyl, or if a perfluoroalkyl group is present at the C-5 of the pyrazoline ring [70].

**Scheme 10:**
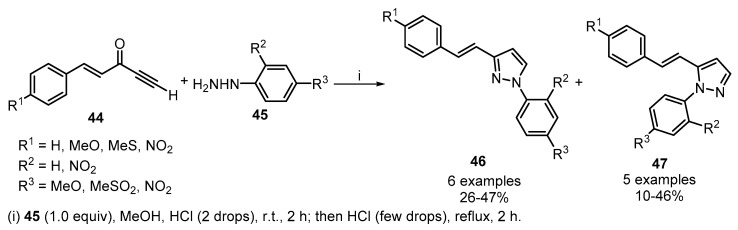
Condensation of acetylenic ketones **44** with aryl hydrazines **45** to produce (*E*)-3(5)-styrylpyrazoles (**46** and/or **47**) [71].

The condensation of acetylenic ketones **44** with aryl hydrazines **45** produces (*E*)-3(5)-styrylpyrazoles (**46** and/or **47**) in fair to good yield. The substitution pattern in these pyrazoles depends on the nature of the substituents and mainly on the reaction conditions (Scheme 10) [71]. When methanol is used as solvent and the reaction is stirred at room temperature for a period prior to the addition of acid and heating, (*E*)-5-styrylpyrazoles **47** were obtained as the major products. However, if acid is present and heat applied from the onset, a mixture of (*E*)-3- and 5-styrylpyrazoles **46** and **47** is obtained. Regioselectivity, in this case, varies from 39:61% to 83:17% of **46**:**47**, depending on the nature of the substituents. For instance, when R^1^ = MeO, R^2^ = H and R^3^ = MeSO_2_, the regioisomer **46** is obtained in higher amount. On contrary, when R^1^ = R^2^ = H and R^3^ = NO_2_, **46** is the minor regioisomer. In the absence of acid, the initial reaction step involves Michael addition of the more basic terminal nitrogen of the hydrazine derivative to the terminal acetylenic carbon to form enamine, which exists in tautomeric equilibrium with the isomeric hydrazone. The cyclization of the hydrazone in the presence of added acid subsequently afforded only (*E*)-5-styrylpyrazoles **46**. The formation of this isomer seems to be promoted by the strong electron-donating and electron-withdrawing effect of the substituents.

**Scheme 11:**
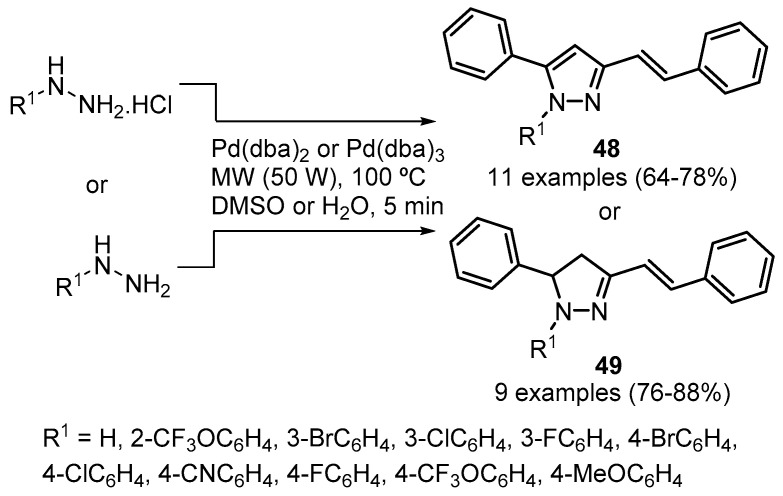
Microwave-assisted synthesis of (*E*)-3-styrylpyrazoles **48** and (*E*)-3-styrylpyrazolines **49** [72].

The microwave-assisted *N*-heterocyclization of metal-diketonic complexes, Pd(dba)_2_ or Pd(dba)_3_ (dba = dibenzylideneacetones) with hydrochloride salts of various aryl hydrazines allows the synthesis of 1-aryl-5-phenyl-3-styryl-1*H*-pyrazoles in a single step and with very good yields (Scheme 11) [72]. Metal-diketones act as both catalyst and coupling partner. The hydrazine substrate and the solvent play an important role in the selectivity. Reaction with phenyl hydrazine hydrochloride in water gave both (*E*)-3-styrylpyrazole **48** and (*E*)-3-styrylpyrazoline **49** in approximately equal amounts. If the substrate is phenyl hydrazine (without hydrochloride salt), (*E*)-3-styrylpyrazoline **49** is the major reaction product. The use of DMSO as a solvent instead of water promotes the formation of (*E*)-3-styrylpyrazole **48** in high yields. 

#### 3.2.2. Intramolecular Oxidative C–N Coupling of Hydrazones

**Scheme 12:**
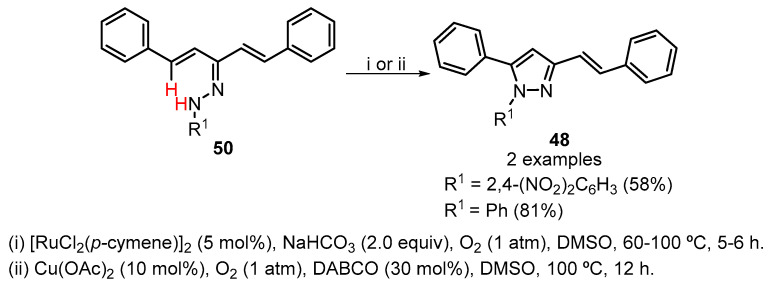
Synthesis of (*E*)-3-styrylpyrazoles **48** by intramolecular oxidative C–N coupling of hydrazones **50** [73,74].

The ruthenium(II)-catalyzed oxidative C–N coupling of 2,4-dinitrophenylhydrazone of (1*E*,4*E*)-1,5-diphenylpenta-1,4-dien-3-one **50** in the presence of oxygen (1 atm) as the oxidant, afforded (*E*)-1-(2,4-dinitrophenyl)-5-phenyl-3-styryl-1*H*-pyrazole (**48**). [RuCl_2_(*p*-cymene)]_2_ (5 mol%) was found to be the best catalyst for this oxidative C(sp^2^)−H amination (Scheme 12) [73]. This catalyst allows C–H bond cleavage via an *o*-metalation process that involves chelation with the nitrogen from hydrazone. Consequently, the formation of a C–N bond is possible via reductive elimination to generate the corresponding pyrazole. Finally, the oxygen promotes oxidation of the Ru(0) species to Ru(II) to complete the catalytic cycle. 

Other oxidative C(sp^2^)−H amination reactions have been reported using copper catalysts. By using Cu(OAc)_2_ (10 mol%) and 1,4-diazabicyclo[2.2.2]octane (DABCO) (30 mol%) in DMSO at 100 °C in the presence of oxygen (1 atm) as the oxidant, (*E*)-1,5-diphenyl-3-styryl-1*H*-pyrazole (**48**, R^1^ = Ph) was obtained (Scheme 12) [74].

#### 3.2.3. Miscellaneous

**Scheme 13:**
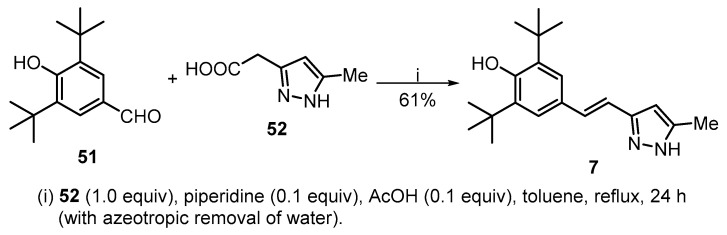
Synthesis of (*E*)-3-styrylpyrazole **7** by Doebner modification of Knoevenagel condensation [33].

The decarboxylative Knoevenagel reaction of aldehyde **51** with 3-methyl-5-pyrazoleacetic acid **52** directly afforded (*E*)-3-styrylpyrazole **7** (Scheme 13) [33].

**Scheme 14:**
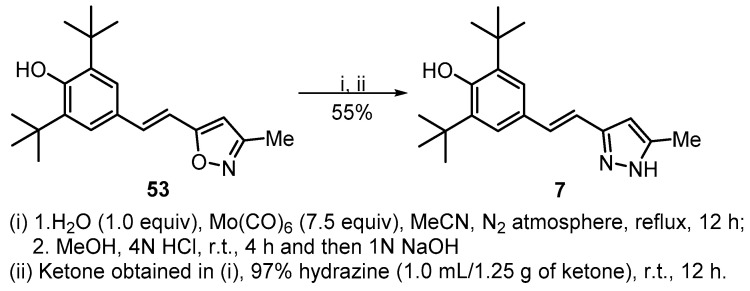
Conversion of (*E*)-5-styrylisoxazole **53** into (*E*)-3-styrylpyrazole **7** by hydrogenolysis [33].

Hydrogenolysis of (*E*)-5-styrylisoxazole **53**, using Mo(CO)_6_ in the presence of water (1.0 equiv), followed by ring closure with hydrazine also produced (*E*)-3-styrylpyrazole **7** [33]. In a first step, a ketone is obtained (Scheme 14, i), which after reaction with 97% hydrazine in acetic acid gave (*E*)-3-styrylpyrazole **7** (Scheme 14, ii).

**Scheme 15:**
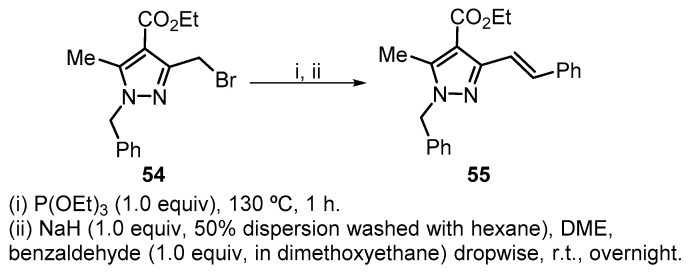
Synthesis of (*E*)-3-styrylpyrazole **55** [75].

Deshayes et al. reported the use of 3-bromomethylpyrazole **54** as template for the synthesis of (*E*)-3-styrylpyrazole **55**. In a first step, this compound was treated with triethyl phosphite, in the Arbusov reaction, affording the corresponding diethylphosphonomethylpyrazole, which then reacted with benzaldehyde in the presence of sodium hydride to give the corresponding (*E*)-3-styrylpyrazole **55** (Scheme 15) [75].

### 3.3. Synthesis of 4-Styrylpyrazoles

#### 3.3.1. Cross-Coupling Reactions

**Scheme 16:**
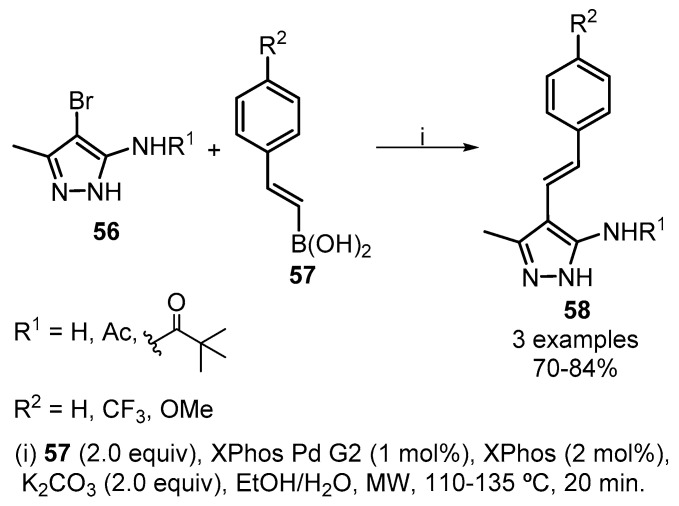
Suzuki–Miyaura coupling of 4-bromo aminopyrazoles **56** and their amides with styryl boronic acids **57** to prepare (*E*)-(4-styryl)aminopyrazoles **58** [76].

**Scheme 17:**
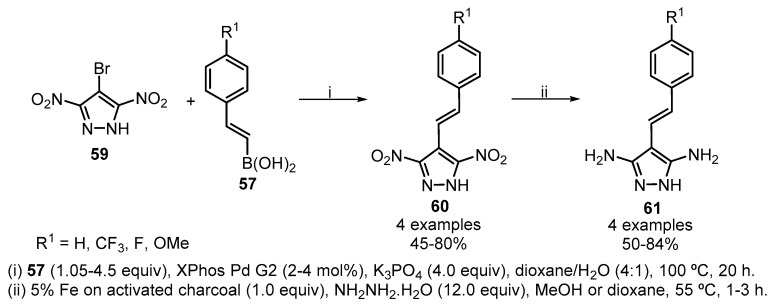
Synthesis of (*E*)-3,5-dinitro-4-styryl-1*H*-pyrazoles **60** and iron-catalyzed reduction of the nitro groups to produce (*E*)-3,5-diamino-4-styryl-1*H*-pyrazoles **61** [77].

(*E*)-(4-Styryl)aminopyrazoles can be accessed in a straightforward way by the Suzuki–Miyaura cross-coupling reaction of 4-bromo aminopyrazoles **56** and their amides with styryl boronic acids **57** (Scheme 16). Using the pre-catalyst palladacycle XPhos Pd G2 and XPhos, in combination with K_2_CO_3_ in a green solvent system (EtOH/H_2_O), under microwave irradiation, Jedináket al. obtained the (*E*)-(4-styryl)aminopyrazoles **58** [76]. Direct comparison of the chloro, bromo, and iodopyrazoles used as substrate in the Suzuki−Miyaura reaction revealed that bromo and chloro derivatives were superior to iodopyrazoles, showing reduced propensity to dehalogenation [76]. Using the same pre-catalyst (XPhos Pd G2), which enabled the coupling with the electron deficient, electron-rich, or sterically demanding boronic acids, Tomanová et al. coupled the 4-bromopyrazoles **59** with styryl boronic acids **57** to prepare the (*E*)-3,5-dinitro-4-styryl-1*H*-pyrazoles **60** (Scheme 17) [77]. The introduction of the electron-deficient nitro groups as masked amino functionalities improved the rate of the oxidative addition step and eliminated the Pd-independent side dehalogenation reaction. Both nitro groups were then converted into amine groups by iron-catalyzed reduction with hydrazine hydrate to give (*E*)-3,5-diamino-4-styryl-1*H*-pyrazoles (**61**).

**Scheme 18:**
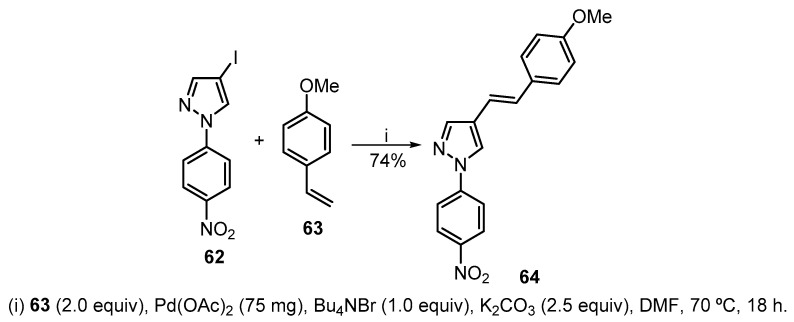
Synthesis of (*E*)-4-(4-methoxystyryl)-1-nitrophenyl-1*H*-pyrazole **64** [71].

**Scheme 19:**
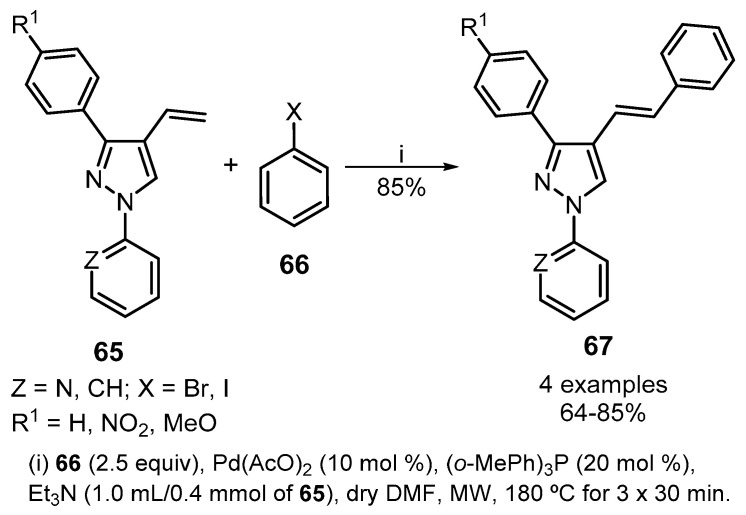
Mizoroki–Heck coupling of 4-vinylpyrazole **65** with halobenzenes **66** to prepare (*E*)-1,3-disubstituted-4-styryl-1*H*-pyrazoles **67** [45].

Starting from 4-iodo-1-(4-nitrophenyl)-1*H*-pyrazole **62** and 4-methoxystyrene **63**, Miller et al. synthesized (*E*)-4-(4-methoxystyryl)-1-nitrophenyl-1*H*-pyrazole **64**, using standard palladium-catalyzed coupling conditions (Scheme 18). Furthermore, 4-bromo-1-(4-nitrophenyl)-1*H*-pyrazole did not react under the same conditions [71]. On the other hand, the Mizoroki–Heck coupling of previously prepared 4-vinylpyrazole **65** with halobenzenes **66**, using Pd(AcO)_2_ (10 mol%) as a catalyst in the presence of a ligand (*o*-MePh)_3_P (20 mol%), afforded the desired (*E*)-1,3-disubstituted-4-styryl-1*H*-pyrazoles **67** [45] (Scheme 19). This reaction was performed under microwave irradiation to obtain the compound in a shorter reaction time.

#### 3.3.2. Cyclocondensation Reaction

**Scheme 20:**
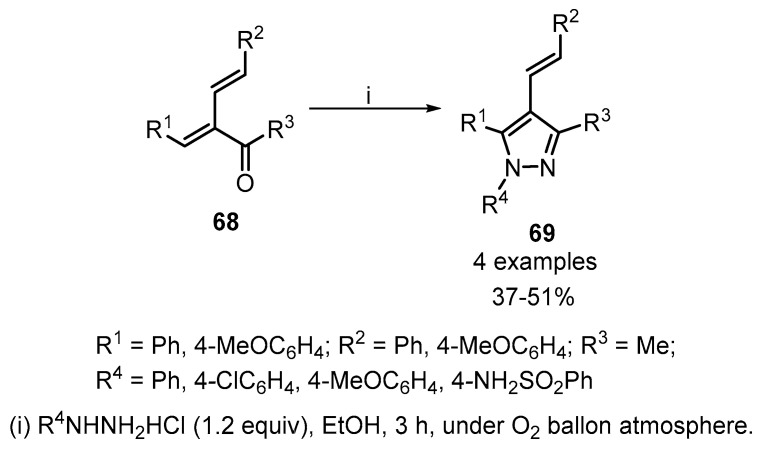
Reaction of α-alkenyl-α,β-enones **68** with arylhydrazines to produce (*E*)-4-styrylpyrazoles **69** [78].

Kim et al. described a convenient method to prepared (*E*)-4-styrylpyrazoles starting from α-alkenyl-α,β-enones **68**, readily accessed from the Morita–Baylis–Hillman reaction. The reaction of **68** with aryl hydrazines in ethanol, in the presence of O_2_, afforded (*E*)-4-styrylpyrazoles **69** (Scheme 20), together with trace amounts of the corresponding 4-arylethylpyrazoles and pyridazine derivatives, which were identified but were not isolated [78].

#### 3.3.3. 1,3-Dipolar Cycloaddition Reaction

**Scheme 21:**
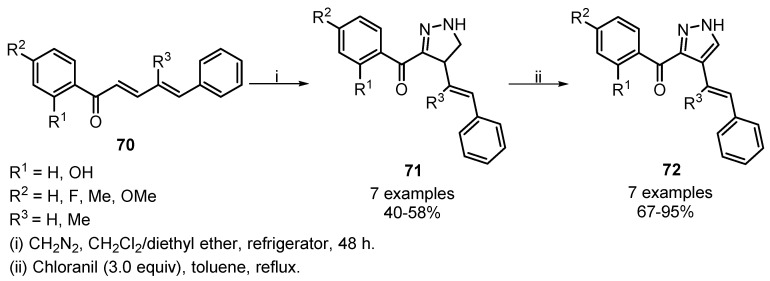
Synthesis of (*E*)-3-benzoyl-4-styryl-2-pyrazolines **71** and 3-benzoyl-4-styryl-1*H*-pyrazoles **72** [79].

**Scheme 22:**
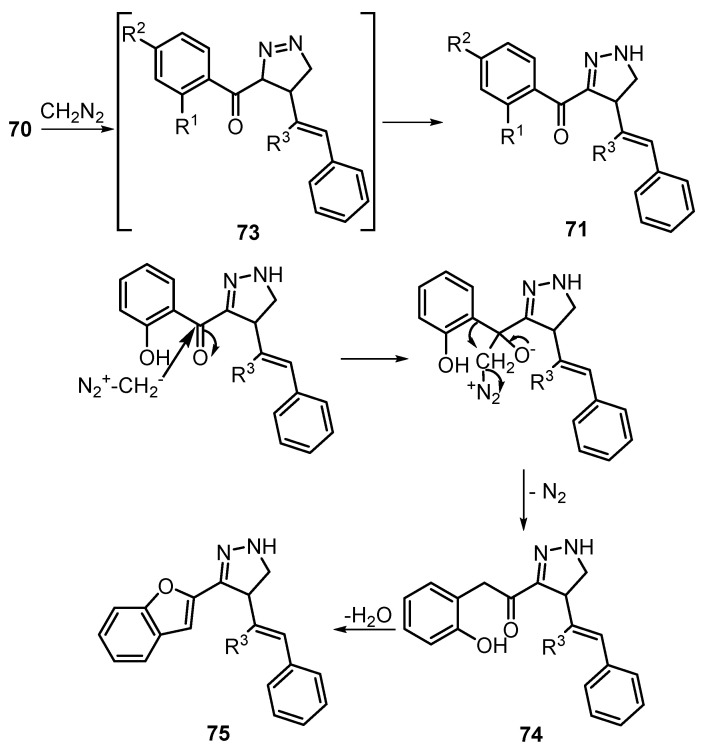
Mechanism of formation of 3-benzoyl-4-styryl-2-pyrazolines **71** and 3-(2-benzofuranyl)-4-styryl-2-pyrazolines **75** [79].

1,3-Dipolar cycloaddition reaction of (*E*,*E*)-cinnamylideneacetophenones **70** and diazomethane, at room temperature or in a refrigerator, lead to the formation of 3-benzoyl-4-styryl-2-pyrazolines **71**. By oxidation with an excess of chloranil, in toluene, pyrazolines **71** were converted into 3(5)-benzoyl-4-styrylpyrazoles **72** (Scheme 21) [79]. The regioselective formation of **71** results from the reaction between the Cα=Cβ double bond of **70** with diazomethane giving rise to 1-pyrazolines **73**, which then isomerize into 2-pyrazoline isomers **71** (Scheme 22). From the reaction between the Cγ=Cδ double bond of **70** with diazomethane, other cycloadducts can be formed; however, their formation was not reported. For some derivatives (R^1^ = H, R^2^ = H and R^3^ = H, Me), small amounts of 3-(2-benzofuranyl)-4-styryl-2-pyrazolines were formed as by-products, as a result of insertion of a methylene group between the carbonyl and the 2-hydroxyphenyl group leading to the formation of compound **74**. Then, the intramolecular reaction of the hydroxy group with the carbonyl carbon and subsequent water elimination from the obtained hemiacetal lead to the formation of the benzofuran ring **75** (Scheme 22) [79].

#### 3.3.4. Reaction of 3-Styrylchromones with Hydrazine Derivatives

**Scheme 23:**
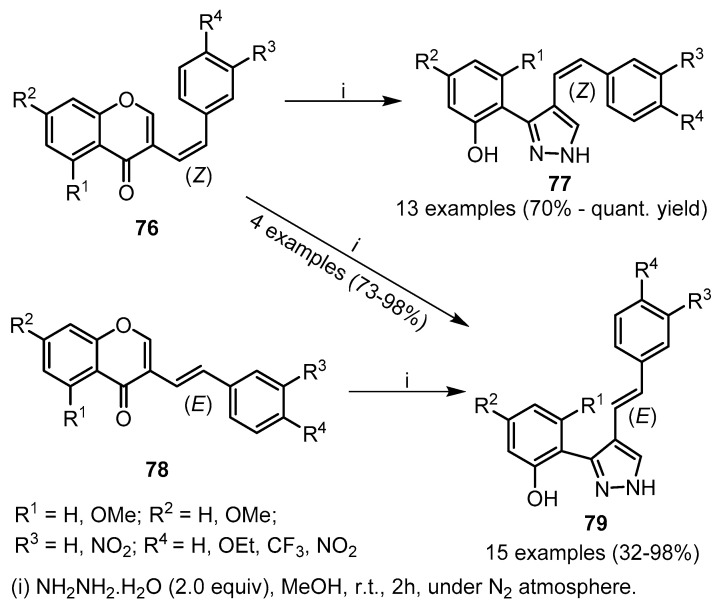
Synthesis of (*Z*)- and (*E*)-3(5)-(2-hydroxyphenyl)-4-styryl-1*H*-pyrazoles **77** and **79** [80,81].

**Scheme 24:**
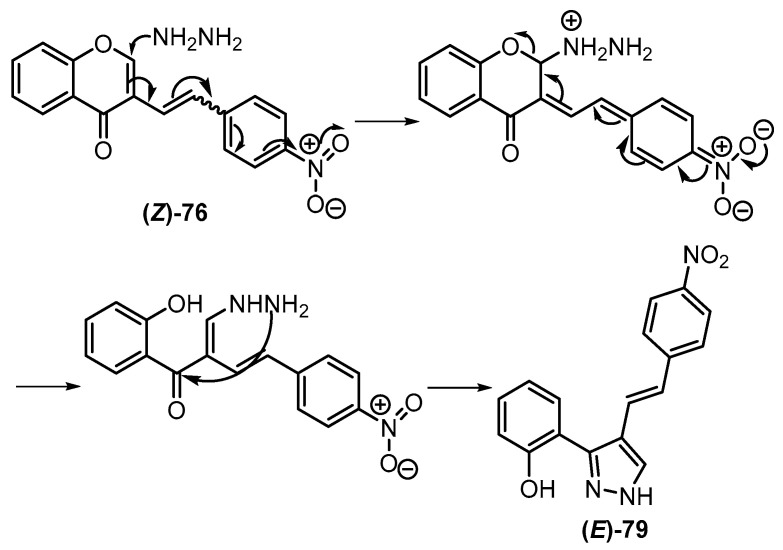
Mechanism of the transformation of (*Z*)-3-(4-nitrostyryl)-4*H*-chromen-4-ones (**76**) into (*E*)-3(5)-(2-hydroxyphenyl)-4-(4-nitrostyryl)-1*H*-pyrazoles (**79**) [81].

The reaction of (*Z*)- and (*E*)-3-styryl-4*H*-chromen-4-ones (**76** and **78**) (also known as 3-styrylchromones) with hydrazine hydrate in methanol, at room temperature, afforded the corresponding (*Z*)- and (*E*)-3(5)-(2-hydroxyphenyl)-4-styryl-1*H*-pyrazoles (**77** and **79**) in 70–94% and 32–98% yield, respectively (Scheme 23) [80]. When the same method was followed to convert (*Z*)-3-(4-nitrostyryl)-4*H*-chromen-4-ones (**76**) into the corresponding pyrazoles, the (*E*)-3(5)-(2-hydroxyphenyl)-4-(4-nitrostyryl)-1*H*-pyrazoles **(79)** were obtained in great yields (>73%) instead of the expected (*Z*)-isomer. These results indicate that the strong electron-withdrawing effect of the *p*-nitro group has an important role in the (*Z*)→(*E*) isomerization during the transformation of (Z)-3-(4-nitrostyryl)-4*H*-chromen-4-ones into the corresponding (*E*)-4-(4-nitrostyryl)-1*H*-pyrazoles. Silva et al. have highlighted the role of the nitro group in the mechanism of formation of the (*E*)-4-styrylpyrazole isomer [81]. After the nucleophilic attack of hydrazine at C-2 of the chromone nucleus, the electronic conjugation moves towards the 4-nitro-3-styryl moiety, allowing the (*Z*)→(*E*) isomerization of the vinylic double bond of the styryl group, to adopt the most stable configuration, with consequent ring opening. Finally, an intramolecular reaction of the hydrazine and carbonyl group led to pyrazole ring formation (Scheme 24). 

Although the biological activity of 4-styrylpyrazoles has rarely been studied, Silva et al. have shown that some derivatives of 4-styrylpyrazoles **77** and **79** with long alkyl chains of ten or twelve carbons on the N-1 or linked at the oxygen of the 2′-hydroxyphenyl moiety present affinity for CB_1_ type cannabinoid receptors in the micromolar range, or even in the nanomolar range, as observed for the (*E*)-4-(4-chlorostyryl)-3(5)-(2-decyloxyphenyl)-1*H*-pyrazole (K_i_ = 53 ± 33 nM) [14].

#### 3.3.5. Miscellaneous

**Scheme 25:**
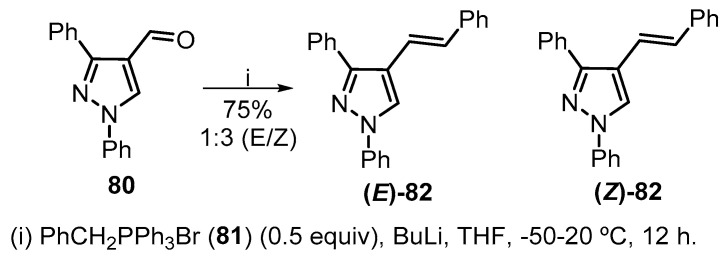
Wittig reaction of 4-formylpyrazole **80** with benzyltriphenylphosphonium bromide **81** to prepare (*E*)- and (*Z*)-4-styrylpyrazoles **82** [45].

The Wittig reaction of 4-formylpyrazole **80** with benzyltriphenylphosphonium bromide (**81**) in BuLi-THF afforded the corresponding (*E*)- and (*Z*)-4-styrylpyrazoles **82** with an *E:Z* ratio of approximately 1:3 (Scheme 25) [45]. 

**Scheme 26:**
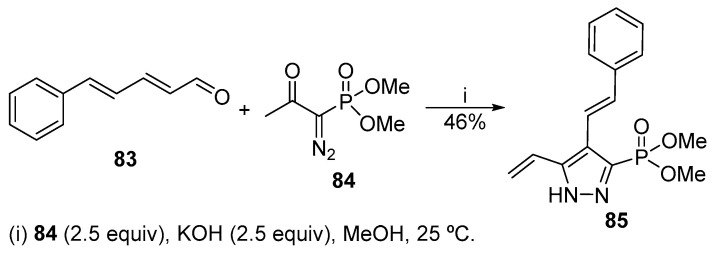
Synthesis of the (*E*)-4-styryl-5-vinylpyrazole **85** [82].

Ahamad et al. described the synthesis of the (*E*)-4-styryl-5-vinylpyrazole **85** by a domino reaction, which involves the 1,3-dipolar cycloaddition of Bestmann–Ohira reagent (BOR) **84** to (2*E*,4*E*)-5-phenylpenta-2,4-dienal **83**, followed by a Horner–Wadsworth–Emmons reaction of the resulting pyrazoline carboxaldehyde and subsequent 1,3-H shift to afford **85**. This methodology has relevant application for the synthesis of 5-vinylpyrazoles (Scheme 26) [82]. 

### 3.4. Synthesis of 5(3)-Styrylpyrazoles

#### 3.4.1. Cyclocondensation Reactions

**Scheme 27:**
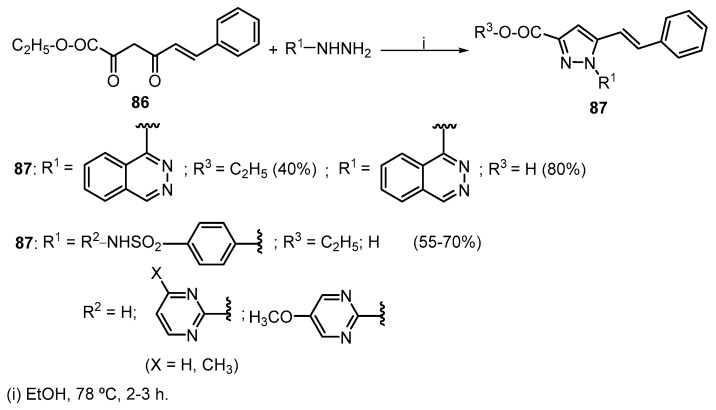
Cyclocondensation of diketoester **86** with aryl/hetaryl hydrazines to prepare (*E*)-5-styrylpyrazoles **87** [83].

One of the first reports about the synthesis of styrylpyrazoles dates back to 1978 [83]. At that time, Soliman et al. described the reaction of diketoester **86** with aryl/hetaryl hydrazines in ethanol to prepare (*E*)-5-styrylpyrazoles **87** (Scheme 27).

**Scheme 28:**
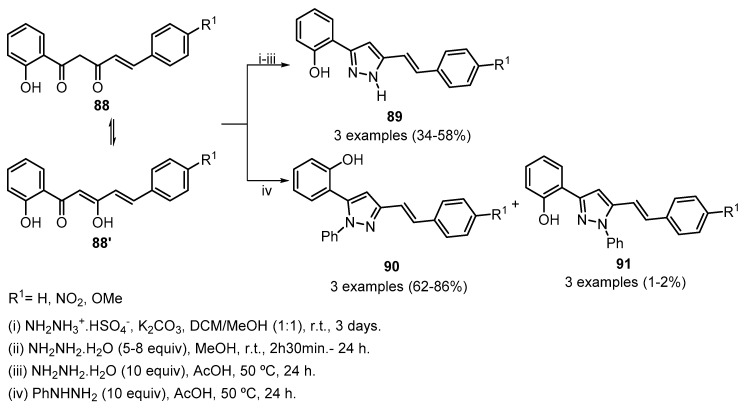
Synthesis of (*E*)-3(5)-(2-hydroxyphenyl)-5(3)-styryl-1*H*-pyrazoles **89**, **90** and **91** [84].

The first approach to the synthesis of (*E*)-3(5)-(2-hydroxyphenyl)-5(3)-styryl-1*H*-pyrazole (**89a**, R^1^ = H) was the treatment of 1-(2-hydroxyphenyl)-5-phenylpent-4-ene-1,3-dione (**88a**, R^1^ = H), which exists in equilibrium with the enolic form (**88a′**), with excess hydrazine (formed by treatment of hydrazinium sulfate with potassium carbonate), added gradually (dropwise) and using a 1:1 dichloromethane (DCM)/methanol mixture as solvent (Scheme 28, i) [84]. Using hydrazine hydrate in methanol at room temperature, compounds **88** were converted into pyrazoles **89** (Scheme 28, ii) [84]. Furthermore, (*E*)-3(5)-(2-hydroxyphenyl)-5(3)-styryl-1*H*-pyrazoles **89** (R^1^ = H, OMe, NO_2_; R^2^ = H) were also obtained starting from **88** by reaction with hydrazine hydrate, in acetic acid (Scheme 28, iii) [84]. Treatment of acetic acid solutions of diketones **88** with an excess of phenyl hydrazine afforded a mixture of two 5-styrylpyrazole isomers **90** and **91**, though pyrazoles **91** were obtained in a vestigial amount (Scheme 28, iv). In fact, the reaction of unsymmetrical diketones and monosubstituted hydrazines, such as phenyl hydrazine, although apparently simple, conceal a complex mechanistic problem. Diketones **88** have two tautomeric forms (**88** and **88′**) and phenyl hydrazine can react initially through NH or NH_2_. When the reaction is carried out in methanol in neutral conditions, a nucleophilic attack of the primary amine (NH_2_) to the more electrophilic position of the diketone (C-1) occurs and only pyrazoles **91** were obtained in low yields. Using acidic conditions (AcOH as solvent), the more basic amine (NH_2_) was protonated and subsequently the nucleophilic attack at the more electrophilic position was through the NH affording pyrazoles **90** [84].

**Scheme 29:**
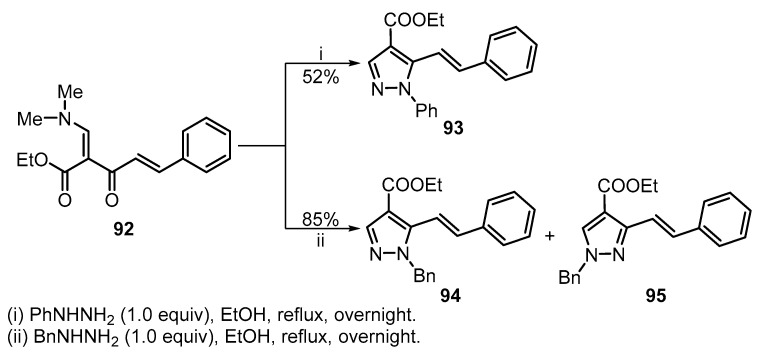
Synthesis of 4-ethoxycarbonyl-5-/3-styrylpyrazoles **93**, **94** and **95** [85].

Deshayes et al. performed the reaction of enamine **92** with phenyl hydrazine in refluxing ethanol, overnight, to produce 4-ethoxycarbonyl-1-phenyl-5-styrylpyrazole **93**. In the same conditions, the reaction with benzyl hydrazine afforded a 3:1 mixture of two pyrazole isomers, the 1-benzyl-4-ethoxycarbonyl-5-styrylpyrazole **94** as the major product together with 1-benzyl-4-ethoxycarbonyl-3-styrylpyrazole **95** (Scheme 29) [85].

#### 3.4.2. Reaction of α,β-Enones with Hydrazines

**Scheme 30:**
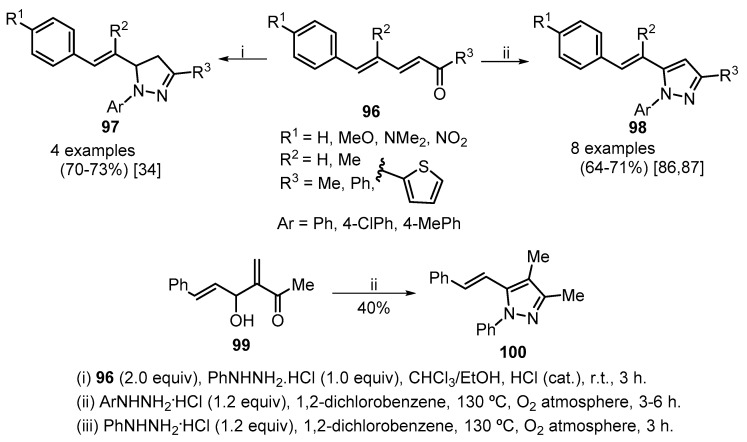
Conversion of α,β-enones **96** and adduct **99** into (*E*)-5-styrylpyrazolines **97** and (*E*)-5-styrylpyrazoles **98** and **100** by reaction with phenyl/aryl hydrazine hydrochlorides [34,86,87].

The reaction of conjugated dienones (or α,β-enones) **96** with phenyl hydrazine hydrochloride in a mixture of ethanol and chloroform, in the presence of a catalytic amount of concentrated HCl, afforded 1,3-diphenyl-5-styryl-4,5-dihydropyrazoles **97** (Ar = Ph) (Scheme 30, i) [34]. No expected dihydropyrazole was obtained in the reaction of 1,5-diphenylpenta-2,4-dien-1-one with phenyl hydrazine due to fast oxidation by atmospheric oxygen affording the corresponding pyrazole [34]. Furthermore, 1-aryl-5-styrylpyrazoles **98** were synthetized by the one-pot reaction of conjugated dienones **96** with aryl hydrazine hydrochlorides in 1,2-dichlorobenzene at 130 °C, under O_2_ atmosphere (Scheme 30, ii) [86,87]. Similar conditions for the reaction of Baylis–Hillman adduct **99** with phenyl hydrazine afforded the styrylpyrazole **100** (Scheme 30) [87]. 

#### 3.4.3. Reaction of 2-Styrylchromones with Hydrazines 

**Scheme 31:**
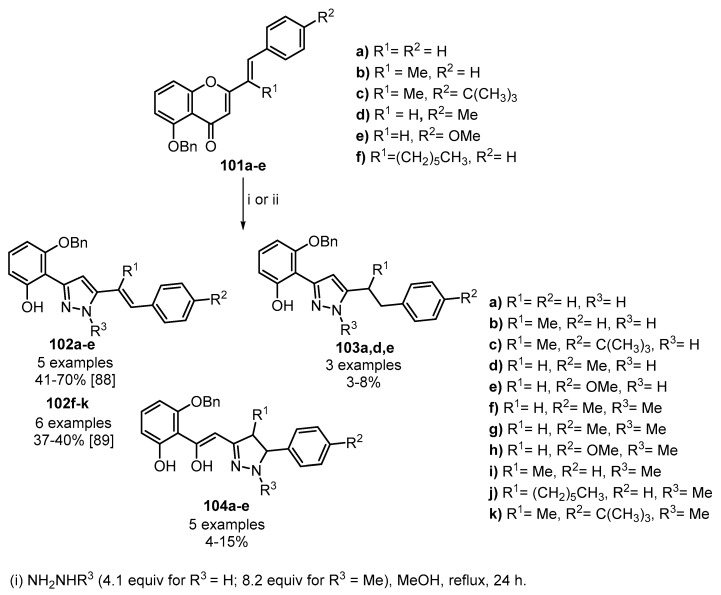
Synthesis of (*E*)-3-(2-benzyloxy-6-hydroxyphenyl)-5-styryl-1*H*-pyrazoles **102** and some derivatives of pyrazoles **103** and **104** isolated as by-products [88].

The reaction of 5-benzyloxy-2-styrylchromones **101** with an excess of hydrazine hydrate in methanol at reflux yielded (*E*)-3-(2-benzyloxy-6-hydroxyphenyl)-5-styryl-1*H*-pyrazoles **102** (Scheme 31). Small amounts of 3-(2-benzyloxy-6-hydroxyphenyl)-5-(2-phenylethyl)pyrazoles **103a**,**d**,**e** and 5-aryl-3-(2-benzyloxy-β,6-dihydroxystyryl)-2-pyrazolines **104a**–**e** were also formed, in addition to the pyrazoles **102a**–**e** [88]. Only one isomer was obtained from the reaction of 2-styrylchromones **101** with methylhydrazine. Due to the hydrogen bond between 6′-OH and *N*-2 in each product, there was the formation of only one tautomer, the 3-(2-benzyloxy-6-hydroxyphenyl)-5-styrylpyrazoles **102f**–**k** and not the corresponding 5-(2-benzyloxy-6-hydroxyphenyl)-3-styrylpyrazole [89].

#### 3.4.4. Wittig–Horner Reaction

**Scheme 32:**
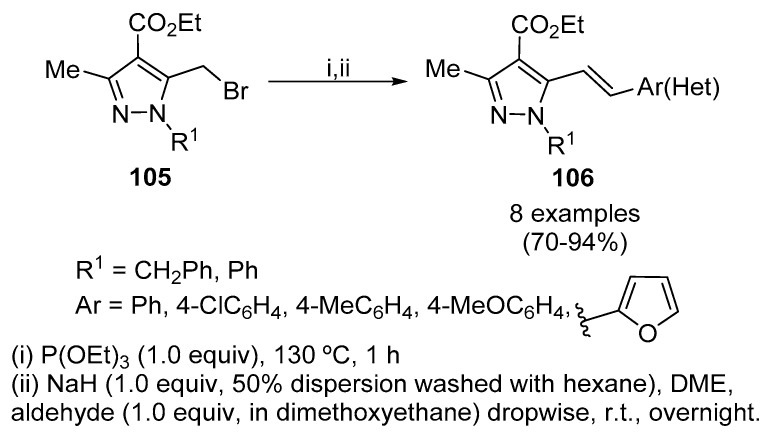
Synthesis of (*E*)-4-ethoxycarbonyl-3-methyl-5-styry-l-substituted pyrazoles **106** [75,85].

Deshayes et al. reported the reaction of phosponic esters, prepared from 1-substituted 5-bromomethylpyrazoles **105** and triethyl phosphite, with substituted benzaldehydes and furfural, in dimethoxyethane, as a method to prepare (*E*)-4-ethoxycarbonyl-3-methyl-5-styryl-l-substituted pyrazoles **106** (Scheme 32) [75,85].

### 3.5. Synthesis of Bis(Styryl)Pyrazoles

#### Cyclocondensation Reaction

**Scheme 33:**
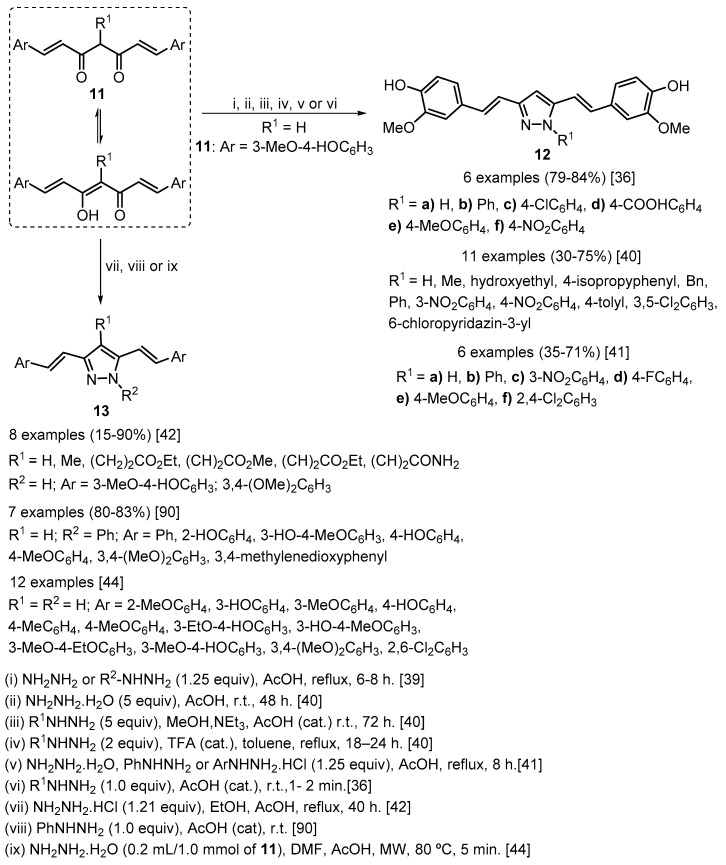
Synthesis of 3,5-bis(styryl)pyrazoles **12** and **13** [36,39,40,41,42,44,90].

Typically, 3,5-bis(styryl)pyrazole curcumin analogue **12a** and *N*-aryl derivatives have been obtained by treatment of curcumin **11** with hydrazine hydrate or aryl hydrazines in ethanol [42] or toluene [40] at reflux for a long reaction time of 24–40 h. Using glacial acetic acid at reflux, the reaction time can be reduced to 6–8 h [33,39,41]. Room temperature reactions have also been reported but required a longer reaction time [40]. In 2015, Sherin et al. reported a solvent-free, mechanochemical method for the synthesis of curcumin **11** derived 3,5-bis(styryl)pyrazoles **12a**–**f** [36] (Scheme 33). The heterocyclization of **11** with hydrazine or hydrazine derivatives was performed with vigorous grinding, using an agate mortar and pestle, at room temperature, in the presence of a catalytic amount of acetic acid. A very short reaction time was necessary, in comparison with the previously referred methods that use conventional heating [39,40,41,42]. The reaction scope seems to be broad since phenyl hydrazine, *p*-methoxy, *p*-chloro, *p*-nitro, and *p*-carboxyphenyl hydrazines gave bis(styryl)pyrazoles **12b**–**f** in good yields (79–84%). One year later, the same authors performed the mechanochemical synthesis of 1-phenyl-3,5-bis(styryl)pyrazoles **13a–f**, varying the substituents present in the aromatic ring of both styryl groups [90]. Recently, Liao et al. described a rapid synthesis of similar 3,5-bis(styryl)pyrazole curcumin analogues **13** by using microwave irradiation conditions [44].

## 4. Transformations of Styrylpyrazoles

Styrylpyrazoles are interesting templates for synthetic manipulation towards new heterocycles. Nevertheless, to date, only a small number of transformations involving the 2-arylvinyl moiety of styrylpyrazoles have been reported in the literature. In this section, we describe the most common transformations of the 2-arylvinyl moiety of styrylpyrazoles.

### 4.1. Transformations of 4-Styrylpyrazoles 

#### Diels–Alder Cycloadditions

**Scheme 34:**
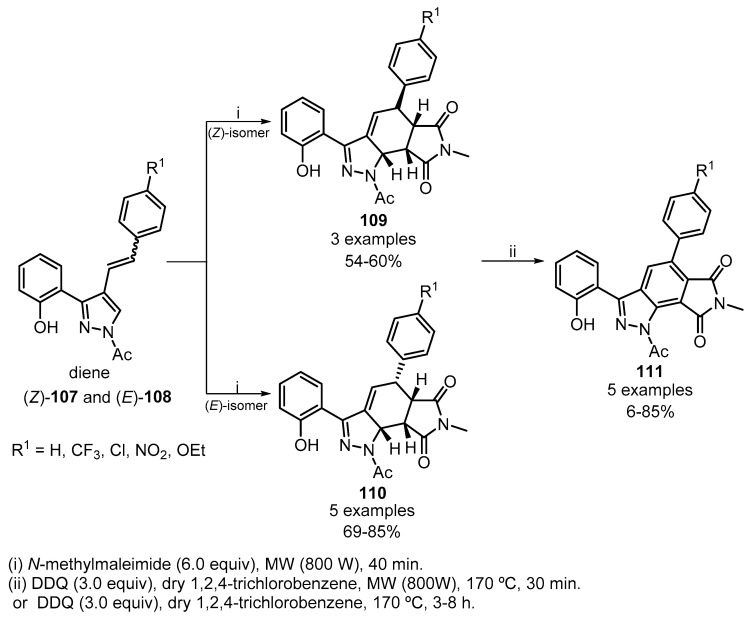
Transformation of 4-styrylpyrazoles **107** and **108** into indazoles **111** [91,92].

**Scheme 35:**
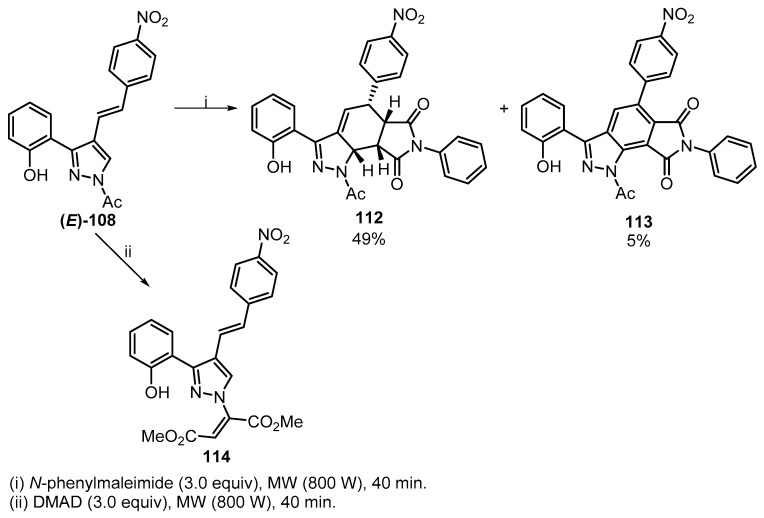
Transformation of (*E*)-4-styrylpyrazole **108** into cycloadducts **112**, **113** and pyrazole **114** [92].

4-Styrylpyrazoles (*Z*)-**107** and (*E*)-**108** can participate in Diels–Alder reactions, as dienophiles, involving the exocyclic (Cα = Cβ) double bond, or as dienes when this double bond is conjugated with the C4 = C3(5) double bond of the pyrazole moiety. Silva et al. obtained the tetrahydroindazoles **109** and **110** in good yields and with high selectivities from the Diels–Alder reactions of (*Z*)- and (*E*)-1-acetyl-4-styrylpyrazoles **107** and **108**, which reacted as dienes, with both dienophiles, *N*-methyl- or *N*-phenylmaleimide, using microwave irradiation (800 W) [91,92]. The expected indazoles **111** were obtained by dehydrogenation of the corresponding adducts with DDQ under microwave irradiation or conventional heating conditions (Scheme 34) [91]. They have also studied the cycloadditions of (*E*)-1-acetyl-4-nitrostyrylpyrazole **108** (R^1^ = NO_2_) with *N*-phenylmaleimide and with dimethyl acetylenedicarboxylate (DMAD). In the reaction with *N*-phenylmaleimide, the cycloadduct **112** was obtained in 49% yield together with the indazole **113**, obtained as a by-product (5% yield). On the other hand, in the reaction with DMAD, a conjugate addition of the pyrazole nitrogen to DMAD occurred with formation of a trace amount of pyrazole **114** (Scheme 35) [91].

### 4.2. Transformations of 3(5)-Styrylpyrazoles 

#### 4.2.1. Diels–Alder Cycloadditions

**Scheme 36:**
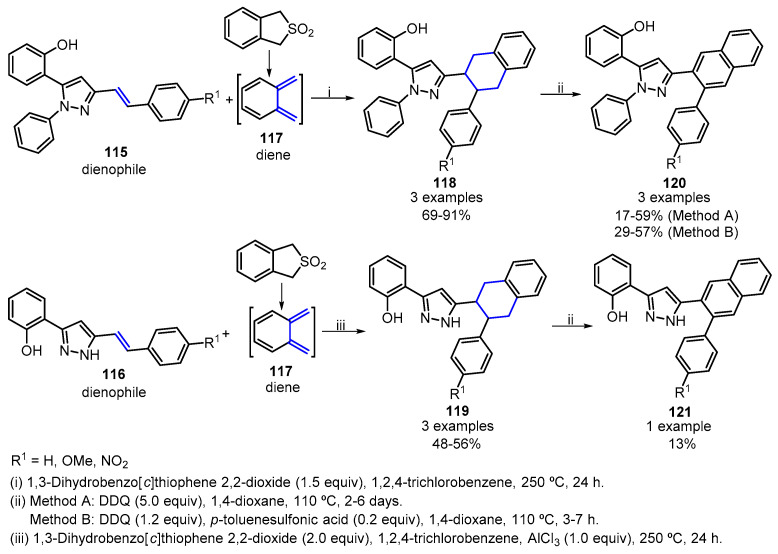
Transformation of styrylpyrazoles **115** and **116** into tetrahydronaphthylpyrazoles **118** and **119** and their oxidation to naphthylpyrazoles **120** and **121** [84].

Starting from 3- and 5-styrylpyrazoles **115** and **116**, Silva et al. developed a synthetic route to prepare naphthylpyrazoles [84]. These styrylpyrazoles, which can behave as dienophiles, undergo a Diels–Alder cycloaddition with *o*-benzoquinodimethane (**117**), the diene, which was formed in situ by the thermal extrusion of sulfur dioxide from 1,3-dihydrobenzo[*c*]thiophene 2,2-dioxide. *N*-Substituted 3-styrylpyrazoles **115** reacted with diene **117** at 250 °C in 1,2,4-trichlorobenzene giving the corresponding 3-[2-(3-aryl-1,2,3,4-tetrahydronaphthyl)]-5-(2-hydroxyphenyl)-1-phenylpyrazoles (**118**) in good yields (69–91%) (Scheme 36, i). The presence of an electron-withdrawing substituent on the *p*-position of the phenyl ring increases the reactivity of the styryl double bond [84]. These authors also performed the Diels–Alder reaction of (*E*)-3(5)-(2-hydroxyphenyl)-5(3)-styrylpyrazoles (**116**) with diene (**117**), but in this case longer reaction times were necessary and the expected cycloadducts were obtained in lower yields (24–48%). The efficiency of this Diels–Alder reaction increased by using a LA, aluminum(III) chloride, which increased the reactivity of the styryl double bond, probably through the formation of an aluminum(III) complex involving the oxygen of the 2′-hydroxy group and the free nitrogen of the pyrazole moiety. Under these conditions, the cycloadduct **119** containing an electron-donating substituent at the *p*-position of styryl moiety was obtained in better yield. On the contrary, the cycloadduct **119** containing the *p*-nitro group as substituent was obtained in better yield without addition of aluminum(III) chloride (Scheme 36, iii) [84]. The formation of naphthylpyrazoles **120** and **121** occurred by dehydrogenation of the corresponding cycloadducts with DDQ (2–6 days). The naphthylpyrazole **120** bearing an electron-donating substituent (R^1^ = OMe) at the *p*-position of the phenyl group linked to the hydroaromatic ring was obtained in a shorter time (2 days) and with better yield (59%) than the other derivatives (R^1^ = H (25%) and R^1^ = NO_2_ (17%)). The presence of the *p*-methoxy substituent stabilizes the carbocation formed through a hydride transfer from compound **118** to DDQ. To increase the yield of this oxidation, *p*-toluenesulfonic acid was added and the products were obtained in a shorter time (2–7 h) and with better yields, especially for the derivative containing the nitro group (R^1^ = NO_2_, 36%) (Scheme 36, ii). The oxidation of cycloadducts **119** was also tried, using both methods (Scheme 36, ii) but only decomposition products were obtained, save the case of 3-(2-hydroxyphenyl)-5-{2-[3-(4-methoxyphenyl)]naphthyl}pyrazole **121** (R^1^ = OMe), which was obtained in low yield (13%, method B) [84].

#### 4.2.2. Electrophilic Intramolecular Cyclization

**Scheme 37:**
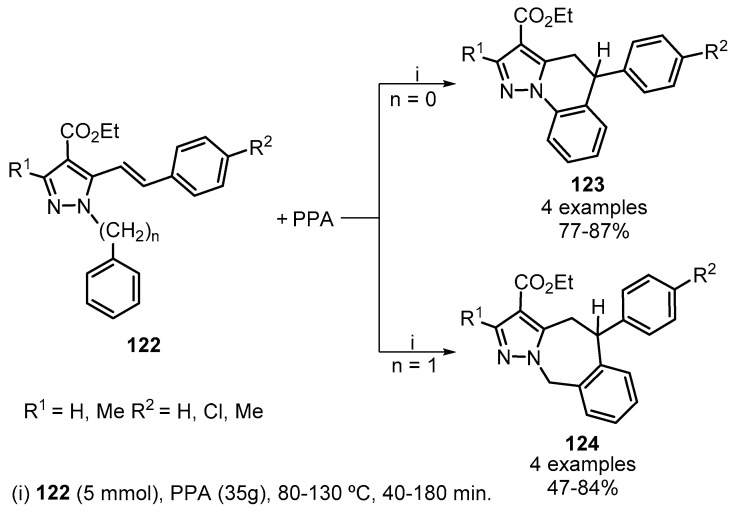
Transformation of 1-benzyl- and 1-phenyl-5-styrylpyrazoles **122** into 5-aryl-3-ethoxycarbonyl-4,5-dihydropyrazolo-type compounds **123** and **124** [85].

C. Deshayes et al. reported the electrophilic intramolecular cyclization of 1-benzyl- and 1-phenyl-5-styrylpyrazoles **122**, in the presence of polyphosphoric acid (PPA), which led to the formation of 5-aryl-3-ethoxycarbonyl-4,5-dihydropyrazolo-type compounds **123** and **124** (Scheme 37) [85]. Both the 5-aryl-3-ethoxycarbonyl-4,5-dihydropyrazolo[1,5-*a*]quinolines (**123**) and 5-aryl-3-ethoxycarbonyl-4,5-dihydro-10*H*-pyrazolo[1,5-*b*][2]benzazepines (**124**) were obtained with very good yields, save for **124** (R^1^ = R^2^ = H).

**Scheme 38:**
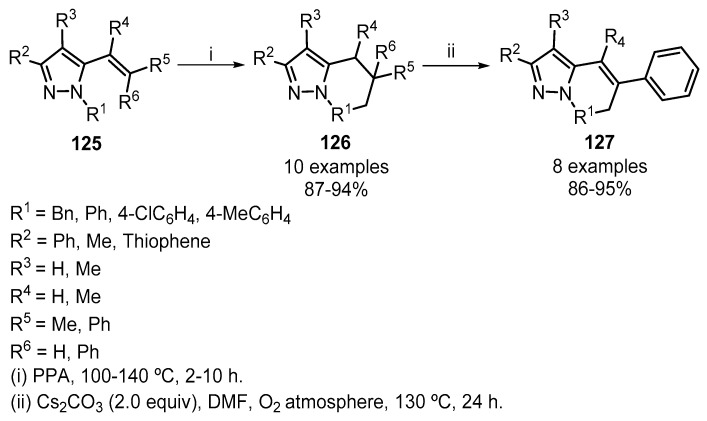
Transformation of 1-aryl-5-styrylpyrazoles **125** into dihydropyrazolo[1,5-*a*]quinolines **126** and their oxidation to pyrazolo[1,5-*a*]quinolines **127** [87].

In 2015, Moon et al. reported the acid-catalyzed intramolecular Friedel–Crafts (IMFC) reaction of several 1-aryl-5-styrylpyrazoles **125** in the presence of PPA to produce dihydropyrazolo[1,5-*a*]quinolines **126** (Scheme 38, i) [87]. It is worth mentioning that the corresponding five-membered ring product was not obtained. This fact can be explained by the higher stability of benzylic carbocation when compared to secondary carbocation formed during the reaction. Moreover, the obtained yields show that the efficiency of the reaction is not significantly affected by the nature of the substituents at the 1-, 3-, 4- and 5-positions of pyrazoles **125**. The IMFC reaction of 1-benzyl-5-styrylpyrazole **125** (R^1^ = Bn) afforded a 2,9-diphenyl-3,3a-diazabenzo[*f*]azulene derivative [87]. The base-catalyzed aerobic oxidation of dihydropyrazolo[1,5-*a*]quinolines **126** afforded the corresponding pyrazolo[1,5-*a*]quinolines **127** (Scheme 38, ii). However, the reaction only occurred when R^5^ = Ph and R^6^ = H [87].

#### 4.2.3. Oxidative Addition Reactions

**Scheme 39:**
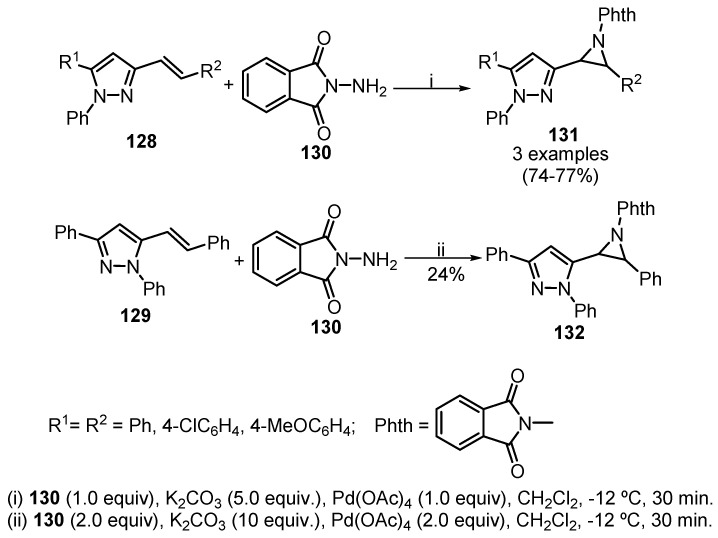
Transformation of (*E*)-3- and 5-styrylpyrazoles **128** and **129** into phthalimidoaziridinylpyrazoles **131** and **132** [57].

In 2004, Ignatenko et al. converted several (*E*)-3- and 5-styrylpyrazoles **128** and **129** into phthalimidoaziridinylpyrazoles **131** and **132** by the oxidative addition of *N*-aminophthalimide (NAPhth) (**130**) to the exocyclic double bond in the presence of lead tetraacetate and potassium carbonate in dichloromethane (Scheme 39). The reaction with 1,5-diphenylpyrazoles **128** afforded the corresponding phthalimidoaziridinylpyrazoles **131** with better yield than with the 1,3-diphenylpyrazoles **129**. Analogous 4,5-dihydropyrazoles were inert in the oxidative addition of NAPhth [57].

## 5. Conclusions

Styrylpyrazoles have shown remarkable biological activities, namely as anti-inflammatory, antimicrobial (antibacterial and antifungal) and antioxidant agents. Among these compounds, the 3,5-bis(styryl)pyrazoles, curcumin analogues, should be highlighted herein due to their significant antioxidant, neuroprotective, antimalarial, antimycobacterial, antiangiogenic, cytotoxic and antiproliferative activities. Additionally, styrylpyrazoles present interesting photophysical properties suitable for metal ion sensing, for the design of energy-transfer-based fluorescent probes and are also good DNA groove binders. Although structure–activity relationship studies have been reported for some styrylpyrazole derivatives, there is a gap in the literature regarding a detailed investigation of the specific properties due to the presence of the styryl group, or the different properties that may appear by exchanging the styryl group position. As far as we know, there are no comparative data between styrylpyrazoles and analogous non-styrylpyrazoles, which are very important in order to understand the role of the styryl moiety in the pyrazoles’ properties.

The synthesis of 1-styrylpyrazoles has been achieved starting from pyrazoles through *N*-cross-coupling reactions with styrylboronic acid or alternatively by the addition of N–H to activated and non-activated alkynes. In turn, the cyclocondensation reaction of different substrates, with hydrazine derivatives, is the most common strategy for the synthesis of 3-, 4-, and 5- styrylpyrazoles. Regarding the substrates, penta-1,4-dien-3-ones, α-oxoketene dithioacetals, benzalacetones, acetylenic ketones, 1,3-diketones and diketoesters are good substrates for the synthesis of 3- and 5- styrylpyrazoles while α-alkenyl-α,β-enones and diketones (curcumin analogues) are good synthons for the synthesis of 4-styrylpyrazoles and bis(styryl)pyrazoles, respectively. Moreover, reactions of 2- and 3-styrylchromones with hydrazine derivatives are often used to prepare 3(5)-styrylpyrazoles and 4-styrylpyrazoles, respectively. More recently, the cross-coupling reactions of halopyrazoles with styrylboronic acids gained relevance as a method to access 4-styrylpyrazoles in a straightforward way.

Only a few examples of transformations of styrylpyrazoles involving the 2-arylvinyl moiety have been reported in the literature. These include Diels–Alder reactions, intramolecular cyclisation reactions and oxidative additions to the exocyclic double bond, and they allow the formation of more complex and very interesting heterocycles such as indazoles, naphthylpyrazoles, 4,5-dihydropyrazolo[1,5-*a*]quinolines, 4,5-dihydro-10*H*-pyrazolo[1,5-*b*][2]benzazepines, pyrazolo[1,5-*a*]quinolines, 2,9-diphenyl-3,3a-diazabenzo[*f*]azulene and phthalimidoaziridinylpyrazoles. Therefore, further studies towards the investigation of novel transformations of styrylpyrazoles are of high interest.
